# DNA methylation of distal regulatory sites characterizes dysregulation of cancer genes

**DOI:** 10.1186/gb-2013-14-3-r21

**Published:** 2013-03-12

**Authors:** Dvir Aran, Sivan Sabato, Asaf Hellman

**Affiliations:** 1 The Department of Developmental Biology and Cancer Research, Institute for Medical Research Israel-Canada, Faculty of Medicine, Hebrew University-Hadassah Medical School, Ein Kerem Campus, Jerusalem 91120, Israel; 2 The Rachel and Selim Benin School of Computer Science and Engineering, Hebrew University of Jerusalem, Edmond J. Safra Campus, Jerusalem 91904, Israel

**Keywords:** Cancer, DNA methylation, distal control elements, enhancers, epigenomics, gene-enhancer pairing, gene regulation, machine-learning

## Abstract

**Background:**

Abnormal epigenetic marking is well documented in gene promoters of cancer cells, but the study of distal regulatory siteshas lagged behind.We performed a systematic analysis of DNA methylation sites connected with gene expression profilesacross normal and cancerous human genomes.

**Results:**

Utilizing methylation and expression data in 58 cell types, we developed a model for methylation-expression relationships in gene promoters and extrapolated it to the genome. We mapped numerous sites at which DNA methylation was associated with expression of distal genes. These sites bind transcription factors in a methylation-dependent manner, and carry the chromatin marks of a particular class of transcriptional enhancers. In contrast to the traditional model of one enhancer site per cell type, we found that single enhancer sites may define gradients of expression levels across many different cell types. Strikingly, the identified sites were drastically altered in cancers: hypomethylated enhancer sites associated with upregulation of cancer-related genes and hypermethylated sites with downregulation. Moreover, the association between enhancer methylation and gene deregulation in cancerwas significantly stronger than the association of promoter methylationwith gene deregulation.

**Conclusions:**

Methylation of distal regulatory sites is closely related to gene expression levels across the genome. Single enhancers may modulate ranges of cell-specific transcription levels, from constantlyopen promoters. In contrast to the remote relationships between promoter methylation and gene dysregulation in cancer, altered methylation of enhancer sites is closely related to gene expression profiles of transformed cells.

## Background

DNA methylation is a key determinant of regulatory chromatin complexes transmitted through cell divisions. Relationships between DNA methylation and gene expression levels were first recognized at gene promoters and CpG islands [1], but were recently observed across the genome [2-6]. In gene promoters, DNA methylation mediates silencing by altering the binding of transcription modulators to their DNA targets [7], but the cause and function of expression-associated methylation away from gene promoters has been elusive. The relationships between aberrant DNA methylation and the altered expression profiles of cancer cells are also not well understood: the predominant pattern in gene promoters is *denovo *methylation of *polycomb*-repressed genes [8-11]. Since these genes are already inactive in the normal tissue and generally remain inactive in the cancer [12,13], it is hard to establish direct contribution to the cancer process [14]. Away from gene promoters, perturbed methylation has been associated with reduced genomic stability [15,16] and global silencing of large chromatin domains [17,18], but the effect on transcription of particular genes remains unknown.

Transcriptional enhancers support tissue-specific expression profiles through physical interactions with gene promoters. Based on analyses of chromatin structures, hundreds ofthousands of transcriptional enhancers were predicted in mammalian genomes (reviewed in [19]). These sites bind chromatin-modulating factors, interact with distal promoters through DNA loops, and demonstrate a unique pattern of DNA methylation [20]. In some particular examples, enhancer sites turned out to be less methylated in cells expressing the controlled genes than in non-expressing cells [21-24]. Taking a more systematic approach, Wiench*et al*. recently revealed activity-dependent methylation in a group of distal enhancers in the mouse [25], and Bock *et al*.[26] demonstrated correlations between enhancer methylation and expression of particular developmental genes during mouse tissue differentiation.

We conducted a methodical analysis of distal DNA methylation sites associated with gene expression in normal and cancerous human cells. The results suggest that enhancer methylation corresponds closely with expression profiles of cancer genes in transformed cells.

## Results and discussion

To explore relationships between DNA methylation and gene expression levels across the genome, we took the following strategy. First, we developed a model for the relationships between methylation in gene promoters and gene expression using a machine-learning algorithm. Then, we applied the derived model to methylation sites away from gene promoters and characterized the chromatin structure and binding profile of the discovered sites. Finally, we explored how the identified sites were altered in cancer.

We analyzed available DNA methylation and gene expression data for 58 human cell types (Table S1 in Additional file 1). The methylation data were produced by two different assays: reduced representation bisulfite sequencing (RRBS) andInfinium HumanMethylation450 BeadChip(Illuminainc., San Diego, CA, USA). Following verification of high agreement between the assays (Figures S1A and S2A, Bin Additional file 1), we combined the two datasets. We then analyzed variation in methylation levels across the panel of cell types (see Materials and methods for detailed description).Sites with methylation levels that did not change across the cell types were eliminated, and the remaining 670, 906 variable methylation sites (VMSs) were used in the study (Figure S1Bin Additional file 1). VMSs in gene promoterswere negatively correlated with expression whereas VMSs in gene bodies were positively correlated with expression (Figure S2 in Additional file 1), as previously reported [6]. The connection with expression levels was most evident at -500 to +2, 000 base pairs relative to transcription start sites (TSSs) (Figure S2A-Cin Additional file 1). Therefore, we used the CpG methylation sites in this range for model development.

### Promoter-based model of regulatory methylation sites

**Figure 1:**
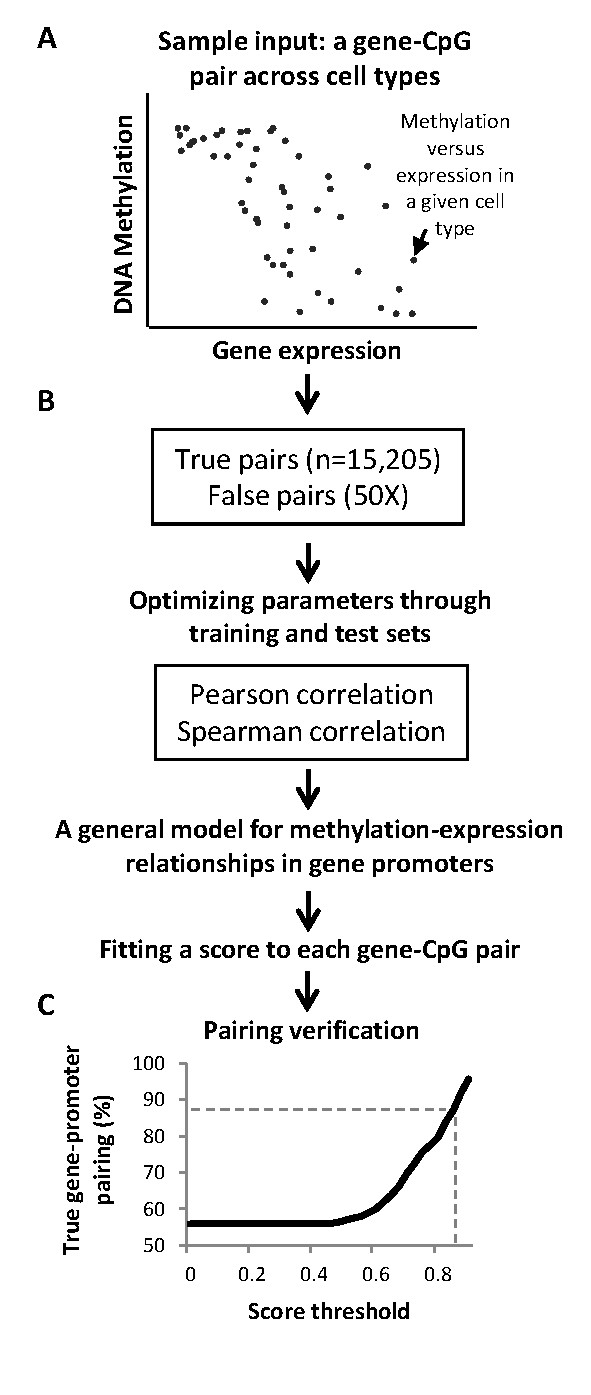
**Promoter-based model of methylation-expression relationships at regulatory sites**. **(A) **A typical gene-CpG sample pair, conveying the methylation of a promoter-based CpGsite in relation to the expression levels of its linked gene across cell types.**(B) **A machine-learning algorithm (SVM-MAP) was trained to distinguish true gene-CpG pairs out of 50-fold excess of false (randomized) pairs. Through rounds of training and test sets, the algorithm optimized parameters of linear (Pearson coefficient) and monotonic (Spearman) correlations to provide the best discrimination between true and false pairs, producing a general model for methylation-transcription relationships in gene promoters. Based on fitting with the learned model,a score was assigned to each gene-CpG pair. **(C) **Rates of successful gene-promoter pairing as a function of thresholds on the scores (null expectation = 50%). At score ≥0.85, the model successfully paired 87.2% ofthe genes to their actual promoters (dashed lines).

As a first step in our study, we tested whether we could couple promoters with the appropriate genes based solely on DNA methylation and gene expression data. We identified 15, 205 VMSs in promoters of 3,434 genes. These gene-CpG pairs provided the 'true' methylation-expression sample inputs (Figure 1A). We then trained a machine-learning algorithm to discriminatethese true gene-CpG pairs out of an excess offalse (randomized)pairs, produced by matching promoter CpGs with genes from other chromosomes. Through rounds of training and test sets, the algorithm optimizes parameters of linear and monotonic correlations between methylation and expression, allowing the best discrimination of the true pairs (Figure 1B). The output was a general model for the methylation-expression relationship in gene promoters (including promoters with or without CpG islands). Based on the learned model, we further produced a score for each of the gene-CpG pairs. At score ≥0.85, the model successfully paired 87.2% ofthe genes to their actual promoters, compared with a null expectation of 50% (Figure 1C). The sensitivity of the test under these conditions was 2.63% (that is, predictions were made for 2.63% of the promoter VMSs), and the false discovery rate was 12.8%. Thus, the developed model successfully paired promoter methylation sites with their genes based on methylation and expression data alone.

### Relating genes to distal regulatory sitesusing DNA methylation

**Figure 2:**
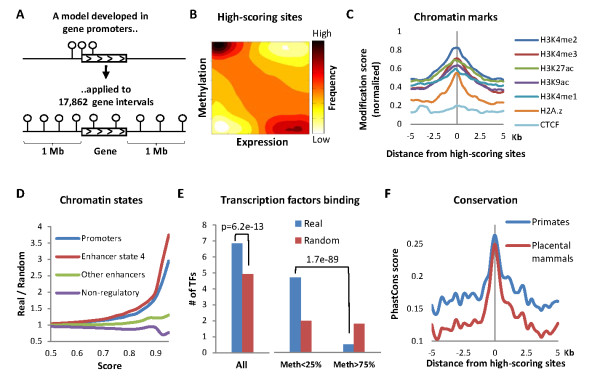
**Mapping and validating distal regulatory sites using DNA methylation**. **(A)**Mapping strategy: a model for methylation-expression relationships in gene promoters was applied to VMSs from 1 Mb upstream through 1 Mb downstream of 17,862 genes. **(B)**Distribution of methylation-versus-expression levels for high-scoring gene-CpG pairs (score ≥0.9, *n *= 2,824). **(C)**Relative enrichment of chromatin factors around the high-scoring methylation sites (*n *= 1,911), excluding sites in the promoters of the associated genes. Data were normalized to 0 to 1 scale.**(D) **Fold enrichment of methylation sites (*n *= 2,824) in actual gene intervals (real), versus the null expectation based on random permutations of gene expression data (random), of chromatin states defined by the ChromHMM algorithm [27].**(E)**Left: Number of transcription factors binding to the high-scoring sites, compared with random expectations. Right:Number of transcription factors binding to unmethylated or methylated enhancers, compared with random expectations. Averages of four cell types for which methylation and binding data were available (GM12878, HepG2, HeLaS3, K562) are shown.Sites in the promoters of the associated genes were excluded.**(F) **Evolutionary sequence conservation around the top-scoring sites. The analyses shown in D and E excluded all sites at ±5 kb from TSSs. TF, transcription factors.

We then extended the analysis to the VMSs residing from one megabases (Mb) upstream of the TSSs through one Mb downstream of the transcription end sites of 17,862 human genes (Figure 2A). We chose to exclude more distant sites to decrease complexity and false positives, and becauseit was previously shownthat distal regulatory elements tend to concentrate in the global vicinities (usually within a few hundredkilobases(kb))of the targeted genes [27,28]. Out of 14,702,075 analyzed CpG-gene pairs, 2,824 pairs obtainedhigh scores (score ≥0.9) according to the model. The distribution of methylation-expression relationships of the high-scoring sites over the cell types is shown in Figure 2B.High-scoring sites were significantly more frequent (*P *<10^-5^) in actual gene intervals than in random intervals (produced by shuffling the expression data of the actual gene with expression data of foreign genes taken from other chromosomes). The entire list of CpG-gene pairs at score >0.75 is given in Additional file 2.

We then sorted out the sites within 5 kb of promoters or alternative promoters of the associated genes. The remaining 1,911 pairs included 486 unique genes corresponding with 1,041 unique distalmethylation regions (we arbitrarily clustered CpGs with <3 kb between them into a single region). On average, we obtained 2.14distal regions and 3.93 methylation sites per associated gene. The following analyses were done on these 1,911 high-scoring CpG-gene pairs.

To confirm that the high-scoring methylation sites wereindeed regulatory sites, we analyzed their chromatin signatures. High-scoring sites were enriched by histone modifications that have been previously associated with transcription activation, including histone 3 lysine 4 methylations, lysine 27 acetylation, lysine 9 acetylation and histone variant H2A.Z, but not with *CTCF *binding (Figure 2C). Further analysis of chromatin states defined by the ChromHMM algorithm (based on various chromatin factors [27]) revealed significant enrichment of the high-scoring sites within promoters and within a particular class (ChromHMM state 4) of strong enhancers (*P *= 2.4e^-15^; Figure 2D). In agreement with this, VMSs were hypomethylated when included in enhancer chromatin, compared to the methylation of the same sites in cells where they were included in non-regulatory chromatin (Figure S3 in Additional file 1). In addition, the highest frequency of hypomethylated sites was observed in state 4 enhancers (Figure S3Cin Additional file 1). We also analyzed the protein-binding capacities of the identified distal sites. The mapped methylation sites bind a larger number of transcription factors than expected. Moreover, unmethylated sites bind more factors than methylated ones (Figure 2E).Finally, we found that the high-scoring methylation sites are evolutionarily conserved (Figure 2F). Together, these findings suggest that distal expression-related methylation sites populate a particular class of transcriptional enhancers, which bind transcription factors in a methylation-depended manner.

### Validation of enhancer-promoter pairing

To validate distal enhancer-promoter interactions, we compared our predictions with a recent comprehensive study of long-range DNA interactions, measured bythe chromosome conformation capture carbon copy (5C) technique in three cell types [29]. We identified enhancer sites (*n *= 318)assessed by both methylation score and 5Cprobes, and determined whether the methylation-based gene-enhancer pairs also reveal physical DNA interactions. Between-assay agreements were markedly increased with methylation scores:at scores ≥0.85, 5C interactions were found in 53% of the cases, compared with 29% of lower score interactions (*P *= 0.021). A representative example of a long-range enhancer-promoter interaction captured by the both assay is shown in Figure S4 in Additional file 1. We also analyzed the frequency of DNA interactions in 21 enhancer-TSS pairs predicted by our assay, versus the interactions of the same enhancers with the other 264 TSSs in the regions (10 kb to 1 Mb from the enhancer site). The frequency of 5C interactions was significantly higher with the TSS predicted by our assay than with the other TSSs (308.5 ±738.2 junction sequence reads in the high-scoring pairs, versus 90.7 ±277.7 reads in the other pairs, *P *= 0.009). Thus, thelarge-scale looping analysis affirms the fidelity of enhancer-promoter pairings based on DNA methylation signals. Given that our study examined many more cell types than the three analyzed in the 5C study, and thus could identify additional enhancers not active in these three cell types, this level of between-assay agreement is probably an underestimation of the actual degree of valid pairs.

Since certain epigenetic profiles may not be fully preserved in cell lines [6], we also confirmed the ability to map enhancer-promoter interactions in freshlyobtained (uncultured) tissue samples. Analysis of seven tissue biopsies from two donors [30]([GSE:30654]) revealed significant intersection (*P *<0.01) with the enhancer-gene pairs observed in the original set of 58 cell types (see Materials and methods for detailed description). Thus, a significant number of the gene-enhancer interactions predicted in cell cultures were replicated in uncultured tissue samples. This analysis also indicates a significant degree of overlapping between gene-enhancer pairing in diverse tissue collections.

### Enhancer methylation defines gradients of cell-type transcription levels from permissive promoters

**Figure 3:**
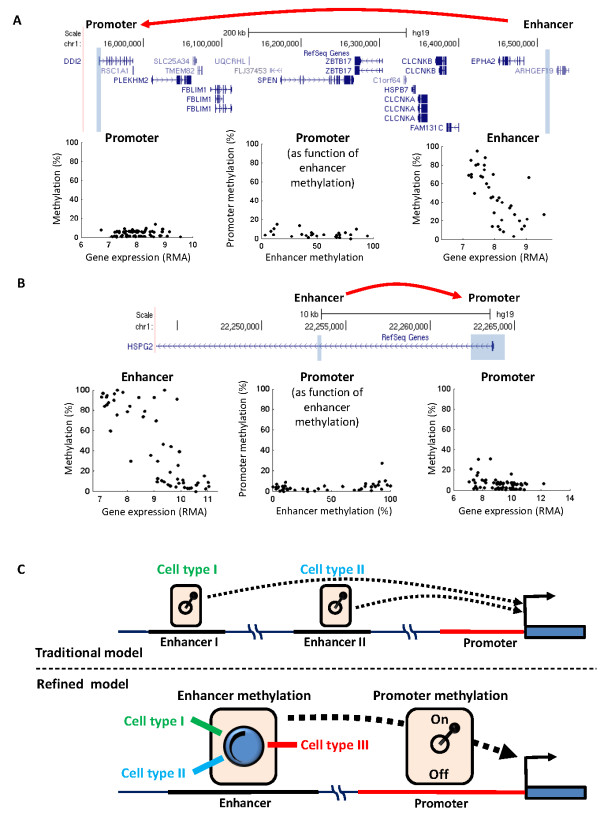
**Enhancer methylation defines gradients of cell-type transcription levels**. **(A)**An example of gene-enhancer pair in the *RSC1A1 *gene region. Gray boxes mark an enhancer methylation site associated with expression of the *RSC1A1 *gene, and the promoter of this gene. The x-y scatters below the map show methylation versus expression across the cell types for the promoter methylation sites (left), and for the enhancer site (right). The middlescatter shows the methylation of the promoter sites versus methylation of the enhancer across the cell types. **(B) **Similar to A, but for an enhancer within the first intron of the *HSGG2 gene*, interacting with *HSGG2*expression. **(C) **Models for enhancer control of cell-type expression levels. Upper panel: The traditional model suggesting that enhancers act like cell-type-specific switches of gene transcription, each of them supports expression in a given cell type (or types). Bottom: A refined model suggesting that even a single enhancer site may functions as a dimmer switch, mediating gradients of transcription levels across many cell types as long as the promoter is unmethylated and thus permissive for transcription. RMA, (Robust Multi-chip Average).

Interestingly, many of the promoters for which we identified high-scoring enhancers were constantly unmethylated across all cell types, regardless of expression levels (Figure 3A,B and Figures S4 and S5 in Additional file 1). This finding does not contradict the bulk of evidence suggesting that methylation sites in and around promoters tend to correlate with expression levels (our model was in fact built based on this general phenomena). Instead, it suggests that in cases of permissive - but not necessarily active - promoters, enhancer methylation may serve as a main determinant of gene transcription levels. Moreover, enhancers tend to show gradual, rather than distinct, methylation levels across cell types, in tight correlation with gradients of expression (note that our model does not enforce these gradients). Enhancers have previously been connected with cell-type-specific transcription patterns. However, it was generally assumed that sets of enhancers, each of them active in a particular tissue or tissues, are needed [31]. According to this model, we would expect that a given enhancer site would be fully methylated in most of the cell types, and fully unmethylated in some. The tendency of the identified sites to show gradual methylation differences between cell types supports an alternative model, in which even a single enhancer site can mediate distinct transcription levels over wide ranges of tissues and cell types (Figure 3C).

### Altered enhancer methylation predicts changes in the expression profiles of cancer genes

**Figure 4:**
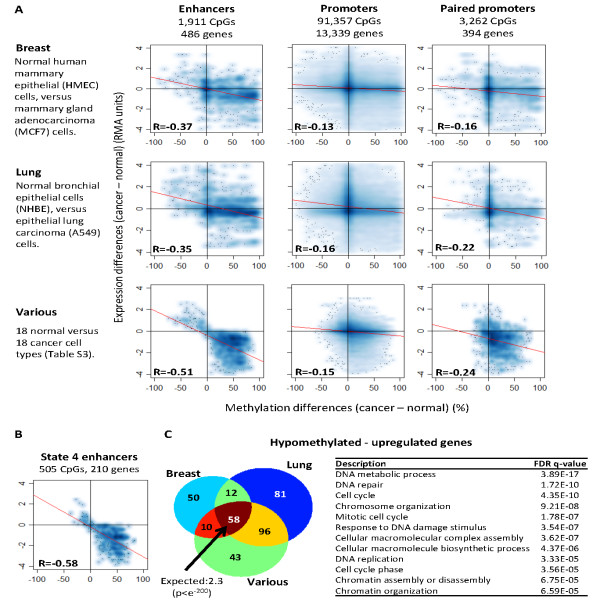
**Altered enhancer methylation predicts changes in the expression profiles of cancer genes**. **(A) **Each of the x-y scatters shows the difference in gene expression between normal and cancer cells as a function of the difference in methylation levels (for example, a difference of +100 indicates that the given site was 0% methylated in the normal cells and 100% methylated in the cancer). The plots on the left are for high-scoring enhancer sites associate with 486 genes,the right plots show the promoters of 394(out of 486) genes that were associated with enhancers, and theplots in the middle show all promoters.Blue dots and clouds donate the frequencies of methylation-versus-expression differences. Linear regression (red lines) and Pearson coefficients (R values) are shown for each scatter. **(B) **Same as in A, but for the 505methylation sites (out of the 1,911) in state 4 enhancers. **(C)**Left:Overlap between the genes (score ≥0.85) in the upper-left quadrants (that is, genes thatwere upregulated by ≥0.25 expression units withdistal enhancers that were hypomethylated by ≥25%) in breast, lung, and the collection of 18 normal versus cancer cell types. The numbers of overlapping genes are indicated. Right: Examples of GO groups that significantly enriched among the 207 genes that were upregulated and hypomethylated in the various cancer types (the entire list is provided in Table S4 in Additional file 1).

Altered histone modifications have recently been demonstrated in enhancers in cancer cells [32]. We therefore explored whether methylation of enhancer DNA is also altered in cancers. We analyzed the methylation levels of the 1,911 distal methylation sites associated with the expression of 486 genes in normal human mammary epithelial cells versus mammary glandadenocarcinoma (MCF7) cells. A subset of the predicted enhancers was hypo- or hypermethylated in the cancer cells compared to the normal cells. The genes associated with hypomethylated enhancers tended to be upregulated in the cancer, and the genes associated with the hypermethylated enhancers tended to be downregulated (Figure 4A, upper row). Moreover, altered gene expression was better correlated with altered enhancer methylation than with altered promoter methylation (R = -0.37 versus R = -0.16, respectively), and the effect of methylation differences on gene expression was significantly higher in enhancer sites than in promoter sites (*P *= 3.55e-^15^). Thus, enhancer methylation is remarkably altered in cancer and, moreover, enhancer methylation is more closely related to changes in gene expression than promoter methylation.

To examine whether these patterns are exclusive to mammary cells, we repeated the analysis in lung epithelia. The analysis of normal human bronchial epithelial cells versus epithelial lung carcinoma (A549) cells revealed similar relationships between enhancer and promoter methylation and gene expression (Figure 4A, middle row). We have further analyzed a set of 18 normal cell types of various tissue origins versus 18 cancers of various tissue origins (Table S2 in Additional file 1). Once again, the methylation of enhancers was drastically altered and was tightly connected with alteration of gene expression, whereas promoters showed smaller alterations and weaker correlation with expression (R = -0.51 versus R = -0.24); *P *<1e^-20^; Figure 4A, lower row). Thus, alteration of enhancer methylation is common to many cancer types, and is associated with substantial changes in gene expression.

Since our enhancers were specifically enriched in the chromatin class defined as ChromHMM state 4 (Figure 2C), and since enhancers in this state showed the highest methylation differences compared to non-regulatory chromatin (Figure S3 in Additional file 1), we hypothesized that state-4 enhancers would show the closest association with changes in gene expression in cancers. Indeed, this group of enhancers showed an even tighter correlation with gene expression levels compared with the entire group of enhancers (R = -0.58 versus R = -0.51; Figure 4B).

We also sought to analyze what characteristics the altered genes might share in addition to association with enhancer methylation. For this, we first analyzed whether the same genes tended to be upregulated or downregulated across cancer types, and then asked whether they belonged to defined gene-ontology (GO) categories. As shown in Figure 4, a majority of the genes were hypermethylated and downregulated, and a smaller fraction of the genes showed the opposite pattern. We found no significant overlap between the genes that were hypermethylated and downregulated in mammary, lung or in the sets of various cell types. These genes also showed no significant GO clustering. Thus, the hypermethylated genes might be cancer-type-specific (for example, cell-type-specific genes that are downregulated in the derived cancer). By contrast, the hypomethylated,upregulated genes significantly repeated between cancer types (Figure 4C, left), and 58 of them appeared in all cancer types examined (*P *<e^-20^, compared with the null expectation of 2.3 overlapping genes; Table S3 in Additional file 1). GO analysis showed that the hypomethylated and upregulated genes were frequently involved in cell growing-related functions (Figure 4C right; an extended list of significant GO clusters is given in Table S4 in Additional file 1). Thus, the hypomethylated enhancers associate with genes that are active in many cancer types and support cell proliferation.

An interesting question raised from the above is whether enhancer methylation may also distinguish between cancer and normally-dividing cells. To examine this, we compared Epstein-Barr virus-immortalizedlymphoblastoid(GM12878)cells, which are rapidly growing in culture but otherwise have normal karyotype and no particular characteristics of cancer cells, with lymphoblastoid (Jukat) cells derived from an acute leukemia.We identified 74 genes that were hypomethylated and upregulated in the leukemia compared to the normal lymphoblastoids. As expected, these were not proliferative genes - the proliferative genes were already active in the dividing normal cells, so they were not expected to show differences compared to the cancer. Instead, this analysis captured genes that were involved in cancer-related processes other than cellproliferation, such as the *PRAME *gene, which is believed to inhibit myeloid differentiation in certain myeloid leukemias[33] (Table S5 in Additional file 1). Of the 74 upregulated genes, 31 were also hypomethylated and upregulated in the sets of normal versus cancer cell types (Figure 4), a significantly higher number than expected by chance (*P *= 7e^-14^). Thus, enhancer hypomethylation may associate with upregulation of genes involved in a variety of cancer-related pathways.

### Enhancer methylation maybe specifically altered in cancer

**Figure 5:**
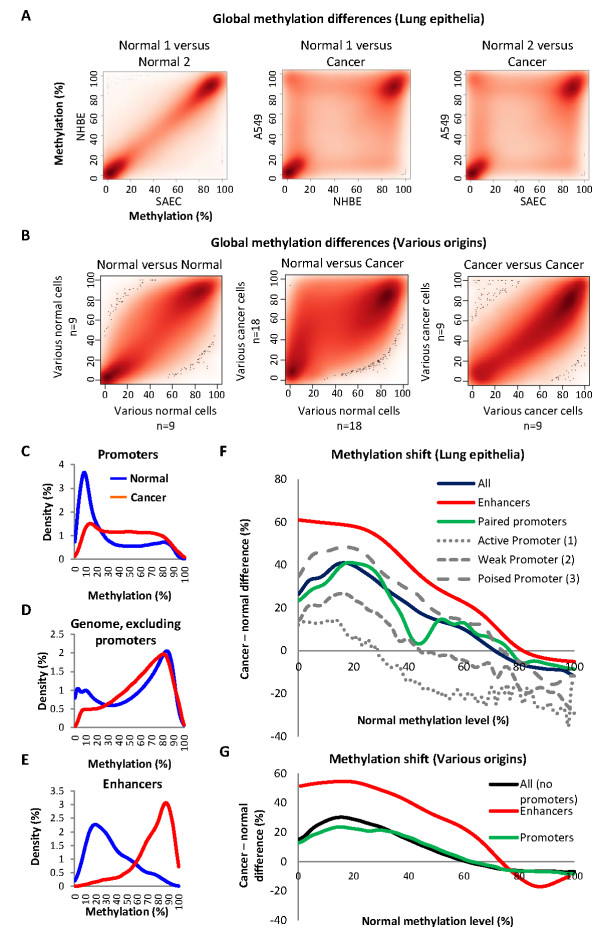
**Enhancer methylation maybe specifically altered in cancer**. **(A,B)**x-y scatters showing the methylation of sites across the genome in a given cell type (or a collection of cell types) versus another cell type (or types).(A)Normal and cancerous lung epithelia: genome-wide methylation of normal versus normal (left) or of cancer versus normal (middle and right) cell types. The given cell types are indicated, as listed in table S2. (B) Normal and cancerous cell types of various tissue origins: genome-wide methylation of nine normal cell types of various tissue origins, versus another nine cell types (left), of 18 cancer versus 18 normal cell types (middle), or of nine cancers versus other cancers (right). **(C-E) **Distributions of methylation levels in the collection of normal and cancer samples shown in B, in promoter sites, in all sites excluding promoters, or in the high-scoring enhancer sites. **(F) **Average gain or loss of methylation in cancer, as a function of the methylation levelin normal cells: for any given level of methylation in the normal cells, the graph shows the average change in the cancer, according to the global trend shown in B. Data are shown for all sites; for sites in the high-scoring enhancers; for sites in the promoters paired with the high-scoring enhancers; or for sites in strong, weak or poised promoters. The analysis is based on methylation levels and chromatin states in human mammary epithelial cells and MCF7 cells shown in A. **(G)**Same analysis as in F, but for 18 normal and 18 cancer cell types shown in B.

Finally, we asked whether the observed perturbations of enhancer methylation in cancers (Figure 4) are fully explained by a global perturbation of the DNA methylation blueprint in cancer cells, or are the outcome of enhancer-specific processes. For this, we first analyzed the overall differences between the methylation of normal and cancer cells. As previously reported, methylation levels along the genomes of normal cells from a given tissue (left scatter in Figure 5A), or even from different tissues (left scatter in Figure 5B), generally resemble each other. Cancer cells, however, are grossly differentiated from normal cells: sites that are normally unmethylated tended to gain methylation, sites that were normally methylated tended to lose methylation, and partially-methylated regions shifted to both directions, accumulating at the extremities of the methylation scale (since larger and smaller shifts must converge at the borders). Due to this effect, cancers are overall more similar to each other than to the normal tissues (Figure 5B). We also analyzed the overall shift in the methylation of particular genomic sections. As expected, promoters, which are generally unmethylated in normal cells, tended to gain methylation in the cancer samples (Figure 5C), while the rest of the genome was slightly hypomethylated (Figure 5D). Surprisingly, high-scoring enhancer sites demonstrated the most dramatic shift between normal and cancer cells: they were partly methylated in the normal cells, and become highly methylated in the cancer (Figure 5E).

With these baseline analyses in hand, we then asked whether any particular group of methylation sites behaved as expected from the global trends, or stand alone. Figure 5F,G shows the average alteration of a given genomic site in cancer, as a function of its methylation level in the normal cells. For example, if a given site was 10% methylated in the normal cells, its average methylation shift in the cancer, based on the analyses shown in Figure 5A,B, was about plus 30% methylation. We found that promoters (green lines in Figure 5E,G) were behaving more or less as expected from the global trend of the genome (black lines). In other words, the alteration of promoter methylation in cancer was similar to the alteration of non-promoter sites with similar normal methylation levels. Looking more carefully into the promoter classes, it appeared that active promoters tended to maintain low methylation against the global trend, whereas poised (*polycomb*-repressed) promoters gained more methylation than expected from the global trend. Yet, the overall alteration of promoter methylation in cancer was relatively close to that expected from the global trend. The shift in the methylationof the high-scoring enhancers, however, was clearly beyond what could be expected from the global trend (Figure 5F,G). Thus, the alteration of the high-scoring enhancer sites in cancer is probably not simply the outcome of the global loss of methylation control in cancer cells.Rather, these sites may be under the effect of a more specific mechanism, such as targeted methylation or demethylation and/or cell selection.

## Conclusions

The mapping of associationsbetween distal regulatory sites and the genes they control is a challenging task, which only recently began to be confronted on the genome-wide scale. Attempts to predict gene-enhancer pairs were based on the profiling of chromatin states or transcription factor binding [27,34], or of long-range DNA looping [28,29].Here, we show that enhancers can be also associated with genes using DNA methylation. In contrast to the above mapping approaches, methylation data are readily available and are highly quantitative, and thus may enhance mapping of gene-enhancer pairing.

We found that distal expression-related methylation sites are abundant in the human genome, co-localizing with enhancer chromatin marks, and are more predictive of expression levels then promoter methylations. While not all distal regulatory sites in the genome must exhibited promoter-like methylation, we showed that a large number of enhancer sites demonstrate reverse correlation between methylation and expression, as in gene promoters.

We have further shown that hypomethylation state is directly related to enhancer activity across cell types (Figure S3 in Additional file 1). The observation that hypomethylated enhancers bind more transcription factors than methylated ones (Figure 2E) suggests a possible mechanism underlying the connection between DNA methylation and enhancer activity. Consistent with this possibility, high-scoring enhancers are particularly enriched within a defined chromatin state (Figures 2D), which is particularly hypomethylated compared to non-regulatory chromatin (Figure S3Cin Additional file 1). Whether this chromatin state holds particularly active enhancers, or perhaps a unique class of methylation-related enhancers, remains to be elucidated.

The range of cell types we analyzed in this study was determined by the availability of methylation and expression data. In addition, the RRBS and Infinium HumanMethylation450 BeadChip methylation data we used provide limited genomic coverage and are strongly biased towards promoters and certain other portions of the genome, while enhancers are not efficiently targeted. Because of this, it is likely that more complete methylomic coverage will expose many additional enhancer-gene pairs. Whole-genome bisulfite sequencing approaches have recently become popular and whole methylome analyses of human tissues are rapidly accumulating. Utilizing our approach, these additional data should allow the production of more comprehensive maps of enhancer-gene pairing across tissues, cell types and conditions.

Unmethylated promoters are permissive for, but do not necessarily determine, transcription initiation. We showed that enhancer methylation associates with cell-type-specific expression levels, even when the promoter is constantly unmethylated (Figure 3). Moreover, enhancer methylation characterizes small (and larger) expression differences. Thus, enhancers are not just on-off switches of cell-type transcription levels, as previously suggested, but may also mediate ranges of expression levels across multiple cell types. In contrast to the traditional model of one enhancer site per cell type, we suggest that a gradient of methylation states at a single enhancer site may direct distinct expression levels in many different cell types (Figure 3C).

In occasional examples, enhancer methylation level has been suggested to be associated with the control of cancer-related genes[35-37]. However, to our knowledge this is the first report on a global association between perturbed enhancer methylation and aberrant expression of cancer genes. We have shown that hypomethylated enhancers associated with the upregulation of many cancer genes controlling various cellular functions (Figure 4C), some of them involved in cell proliferation and some in other cancer-related processes. Moreover, many of these hypomethylated genes were found in most cancer types examined, suggesting a pan-cancer mechanism. However, the larger group of hypermethylated enhancers seemed to target cancer-type-specific genes. Given the limited genomic coverage of this study, many additional cancer-related enhancers are expected in the genome.

To date, almost all studies of cancer-related methylation have focused on gene promoters and CpG islands. Among these, the predominant event in cancers is hypermethylation of *polycomb*-repressed promoters [9-11]. This hypermethylation does not directly affect expression levels, as the associated genes are inactive in the normal tissue and generally remain inactive in the cancer(although it may limit the potential for re-activation of silenced genes in the cancer). Here, we established a very different occurrence in the other large group of regulatory sites - the transcriptional enhancers. These sites are drastically altered in cancers, to both hypo- and hypermethylation, and are closely related to substantial modifications in the expression levels of cancer-related genes (Figure 4). Moreover, their aberrant methylation in cancers might derive from targeted methylation or demethylation or from selection of the altered cells (Figure 5). Whether targeted or selected, aberrant enhancer methylation may be involved in important events during cancer development.

We have uncovered a class of distal methylation sites that closely describe cell-type transcription levels. These sites reside in a particular subclass of transcriptional enhancers and are associated with cell-type-specific enhancer activity, possibly through communication with the binding of transcription factors. Methylation levels of these enhancers associate with gradual expression differences across cell types, even when the linked promoters are consistently unmethylated across the cell types. The radical changes in methylation of these sites in cancer is beyond that expected from the general profile of the cancer methylome, and may reflect specific targeting of the methylation and demethylation machinery to these sites, and/or functional contribution to tumor development. Further analyses of these sites may provide crucial information about paradigms of gene expression control in normal and cancerous cells.

## Materials and methods

### Imported datasets

The following datasets were downloaded from the ENCODE website [38]:

1. DNA methylation data for 58 cells types, for which expression data were also available. Methylation data were the average of two RRBS experiments (52 of the cell types, as detailed in Table S1 in Additional file 1) or Infinium HumanMethylation450 BeadChip data (36 of the RRBS cell types plus additional six cell types for which only BeadChip data were available).

2. Expression data for the above cell types produced by the Affymetrix Human Exon array. Gene-level expression data for 17,862 genes was extracted from the raw data following Robust Multi-chip Average normalization.

3. Histone chromatin immunoprecipitation (ChIP)-sequencing peaks and ChromHMM annotation data available for six of the above cell types, including K562, human mammary epithelial cells, human skeletal muscle myoblasts, normal human lung fibroblasts, GM12878 and HepG2 cells.

4. ChIP-sequencing peaks data for 122 DNA binding factors in various cell types.

Human whole-genome methylation data were from Lister *et al. *[39]. Genomic locations are according to human genome version hg19 of the human genome. Gene sizes, start and end sites, promoters and alternative promoters are according to the RefSeq database (National Center for Biotechnology Information, Bethesda, MD, USA). Methylation and expression data for somatic tissues of two individuals are from [30]([GSE:30654] patients #1 and 2).

### Data filtering and categorization

VMSs were CpGs with standard deviation >5 after omitting outliers utilizing the Thomson's Tau method (alpha = 0.1%). A 'sample' (gene-CpG pair) was the two-dimensional matrix describing the methylation levels of a given CpG site as a function of the expression levels of a given gene across the cell types. For the RRBS data,only methylation sites that were sequenced at least 10times in at least 60% (80% for the Support Vector Machine (SVM) algorithm) of the cell types were included.

### Machine learning

The following fourfeatures were extracted for each CpG-gene sample: positive Pearson coefficient (0 if negative);negative Pearson coefficient (0 if positive); positive Spearman coefficient (0 if negative); and negative Spearman coefficient (0 if positive). Based on these features, we learned a model distinguishing false from true samples, applying a machine-learning algorithm. The SVMmap algorithm is a SVM algorithm for predicting rankings. It performs supervised learning using binary labeled training examples, with the goal of optimizing mean average precision (MAP) [40]. We used SVM-MAP for ranking the CpG-gene samples in 3,434 gene queries (RRBS data in 52 cell types) containing 15,205 true samples and a 50-fold excess of randomly selected false samples, where the goal wasfitting a model that ranks the true samples above the false samples. The regularization parameter was optimized by dividing the gene queries into training and test sets (4:1 ratio) and performing 10-fold cross-validation. Query *P *-values (the probability of obtaining the top ranked true sample by chance) were calculated for the test set queries as follows:

We denote$X={\text{min}}_{\text{T}}\left(\text{rank}\right)$, where  Trepresents the true samples. The event that X=x is equivalent to selecting$\left|T\right|-1$ samples from {x+1,x+2,…,n}. Therefore the probability of this event can be obtained by the hypergeometric distribution:

$$\text{Pr}\left(X=x\right)=\frac{\left(\begin{array}{c} n-x\\ \left|T\right|-1 \end{array}\right)}{\left(\begin{array}{c} n\\ \left|T\right| \end{array}\right)}$$

And the probability that$X\leq {\text{min}}_{\text{T}}\left(\text{rank}\right)$is:

$$\text{Pr}\left(X\leq x\right)=\overset{x}{\underset{i=1}{\sum }}\frac{\left(\begin{array}{c} n-i\\ \left|T\right|-1 \end{array}\right)}{\left(\begin{array}{c} n\\ \left|T\right| \end{array}\right)}$$

The resulting model for the methylation-expression relationship consisted of the following learned features: positive Pearson coefficient = -0.0058, negative Pearson coefficient = 0.0104, positive Spearman coefficient = -0.0038, and negative Spearman coefficient = 0.0088.

### Applying the learned model to the datasets

The model learned in gene promoters was applied to all CpGs in 17,862 gene intervals (1 Mb upstream, gene-body, 1 Mb downstream to each gene), resulting in 14,702,075 CpG-gene pairs.The scores were normalized to a 0 to 1 range(c∈Ca CpG site, g∈G a gene):

$$Score\left(c,g\right)=\frac{\langle model,features(c,g)\rangle -min\left(s\left(C,G\right)\right)}{\max\left(s\left(C,G\right)\right)-min\left(s\left(C,G\right)\right)}$$

### Data analyses

#### Frequency map of high-scoring sites

The distribution of methylation-expression relationships of the high-scoring sites over the cell types is a contour representation of the model learned to distinguish true from random pairs. The frequency map shown in Figure 2B is a representation of the corresponding scatter plot (SVM-MAP for 25 × 25 grids of high-scoring pairs), with smoothing of each point around a sphere (radius = 3).

#### Enrichment of chromatin marks around the high-scoring sites

ChIP-seq data ('modification score') were downloaded from the ENCODE website, averaged across the analyzed cell types, normalized to a 0 to 1 scale, and averaged across the high-scoring methylation sites. Loess smoothing with a span of 10% was applied to the presented data.

#### Enrichment of chromatin states around the high-scoring sites

ChromHMM states were identified for the CpG sites across the cell types. (A state was called when a given site was found in a given state in at least one of the cell types. Sites may be related to more than one state). The actual number of sites in a given chromatin category and a given score level ('real'), was divided by the number obtained from shuffling the expression data between genes 10 times ('random'). *P *-values were calculated based on the normal distribution of the shuffled data.

#### Binding of transcription factors to the high-scoring sites

ChIP-sequencing peaks data for 122 DNA binding factors in fourcell types(GM12878, HepG2, HeLaS3, K562 were downloaded. For every high-scoring site we counted the number of factors that bind, and averaged across the cell types. Random expectation and real versus random *P-values *were obtained as above. The *P *-value for the difference between methylated and unmethylated sites was calculated using the Wilcoxon rank-sum test for difference between averages.

#### Conserved sequences around the high-scoring sites

PhastConsdata (phastCons46wayPrimates and phastCons46wayPlacental) were downloaded from the University of California, Santa Cruz genome browser and plotted around the high-scoring sites.

#### Agreement with gene-enhancer pairing by long-range chromatin interactions

From 5C data produced at the University of Massachusetts in threecell lines (Gm12878, HeLA-S3, K562) we sorted out the enhancer sitesthat were probed by the 5C, for which we also have at least one CpG site available to our analysis at±500 base pairs from the probed enhancer. We then located the TSS that obtained the highest score in relation to the enhancer CpG. This yielded 318 interactions, at distances of 10 kb to 1 Mb between the CpG side and the TSS side. We then counted the number of cases in which the maximum number of interaction sequence reads (average of two repeats over three cell types) washigher than the average interaction read number (98.23 reads). *P *-values were obtained by comparing the binomial probability of the agreement in high versus low scoringsites. We also compared the number of 5C interaction reads inhigh-scoring (score≥0.85) enhancer-TSS pairs, versusthe number interactions between these enhancers and other TSSs at 10 kbto 1 Mb from the enhancer sites. The *P *-value of the difference was calculated by the Wilcoxon Rank-Sum test.

#### Replication in uncultured samples

Methylation and expression data for bladder, lymph node, ureter, lung, stomach, skeletal muscles and adipose tissue biopsies of twoindividuals were downloaded from a recently-published dataset [GSE:30654]. Out of the enhancer-promoter interactions that were predicted in the original set of the 58 cell types, 876 were also assessable in the new dataset (that is, methylation data were available for the relevant CpG sites, and expression data were available for the interacting genes). We re-scored these gene-CpG pairs, using our model, in the new dataset. If the interactions that were predicted in the 58 cell types were not relevant to interactions in the uncultured tissue samples, than we expected that 20.5 ±4.5out of the 876 would (by chance) obtain high-scoring (score>0.9) interactionsin individual #1, and 19.9 ±3.5in individual #2. Instead, we observed 29 in individual #1 (*P *= 0.0145) and 28 (*P *= 0.0084) in individual #2. The *P-*value (normal distribution, two-sided) indicated in the result and discussion section is for the average of both individuals (expected = 20.21±3.206, observed = 28.5 interactions, *P *= 0.0098).

#### Alteration in cancer

Differences betweennormal and cancer cells were measured by Robust Multi-chip Average expression units and by methylation percentages. The distributions of methylation and expression differences between normal and cancer samples (Figure 4) were smoothed (color density representation) using a kernel density estimate (transformation function = x^3). Linear regressions were performed (red lines and Pearson coefficients in Figure 4). The *P-*value of the frequencies of signals in the upper-left and lower-right quarters, in enhancers versus promoters, were calculated using normal distribution of two-proportion z test. *P *-valuesof the overlap between genes in the cancer types were calculated based on the expectation from random intersections between the groups.

#### Gene ontology analysis

Analysis of GO terms of thehypomethylatedupregulated genes was done with the GOrilla tool [41], using the list of high-scoring genes as a background.

## Abbreviations

5C: chromosome conformation capture carbon copy technique; ChIP: chromatin immunoprecipitation; GO: gene ontology; MAP: mean average precision; kb: kilobase pairs; Mb: megabase pairs; RRBS:reduced representation bisulfite sequencing; SVM: Support Vector Machine; TSS:transcription start site; VMS: variable methylation site.

## Competing interests

The authors declare that they have no competing interests.

## Authors' contributions

DA participated in the design of the study, collected and prepared the data, developed the promoter-based model, performed the analyses, and helped to draft the manuscript. SS aided in the development of the promoter-based model. AH conceived and supervised the study and wrote the manuscript.All authors read and approved the final manuscript.

## Supplementary Material

Additional file 1 **Supplementary tables and figures. Table S1**. Human cell types and DNA methylation data that used in the development of the promoter-based model. **Table S2**.Normal and cancer cell types used in the study. **Table S3**. Genes that are hypomethylated and upregulated in the examined cancer types, compared with normal cells.**Table S4**. Enriched GO groups among the genes that were hypomethylated and upregulated in various cancers.**Table S5**.Hypomethylatedupregulatedgenes in acute leukemia.**Figure S1**.The overall structure of the DNA methylation data. **Figure S2**. Methylation levels around TSSs as function of gene expression levels.**Figure S3**.Genomic sites are hypomethylated when marked as enhancer chromatin, compared with the methylation of the same sites in non-regulatory chromatin.**Figure S4**.An example of a long-range enhancer-promoter interaction captured by both methylation-based gene-enhancer pairing and long-range chromatin interactions assessed by the 5C technique.**Figure S5**.Representative examples of methylation-based gene-enhancer pairing. Click here for file

Additional file 2 **Data file**. A CSV file (3.3 MB) denoting CpG position and gene symbols for the 118,417 CpG-gene pairs that obtained score >0.75. Click here for file
