# Supplementary material for: DNA methylation of distal regulatory sites characterizes dysregulation of cancer genes
Source: Genome Biol. 2013 Mar 12;14(3):R21. doi: 10.1186/gb-2013-14-3-r21 (PMC4053839; doi:10.1186/gb-2013-14-3-r21)
Supplement: Additional file 1 — Supplementary tables and figures. Table S1. Human cell types and DNA methylation data that used in the development of the promoter-based model. Table S2.Normal and cancer cell types used in the study. Table S3. Genes that are hypomethylated and upregulated in the examined cancer types, compared with normal cells.Table S4. Enriched GO groups among the genes that were hypomethylated and upregulated in various cancers.Table S5.Hypomethylatedupregulatedgenes in acute leukemia.Figure S1.The overall structure of the DNA methylation data. Figure S2. Methylation levels around TSSs as function of gene expression levels.Figure S3.Genomic sites are hypomethylated when marked as enhancer chromatin, compared with the methylation of the same sites in non-regulatory chromatin.Figure S4.An example of a long-range enhancer-promoter interaction captured by both methylation-based gene-enhancer pairing and long-range chromatin interactions assessed by the 5C technique.Figure S5.Representative examples of methylation-based gene-enhancer pairing. [file gb-2013-14-3-r21-S1.PDF]

Table S1. Human cell types and DNA methylation data that used for the development of the promoter-based model.

Cell	Description	Culture	RRBS	450K
**A549**	Epithelial cell line derived from a lung carcinoma tissue	Cancer derived line		■
**Ag04449**	Fetal buttock/thigh fibroblast (apparently healthy)	Untransformed line	■	■
**Ag04450**	Fetal lung fibroblast (apparently healthy)	Untransformed line	■	■
**Ag09309**	Adult toe fibroblast from apparently healthy 21 year old	Untransformed line	■	■
**Ag09319**	Gum tissue fibroblasts from apparently heathly 24 year old	Untransformed line	■	■
**Ag10803**	Abdominal skin fibroblasts from apparently heathly 22 year old	Untransformed line	■	■
**AoSMC**	Aortic smooth muscle cells	Cell line	■	
**Astrocy**	Astrocytes	Cell line	■	
**Be2_c**	Neuroblastoma	Cancer derived line	■	■
**Bj**	Primary skin fibroblast	Untransformed line	■	■
**Caco2**	Colorectal adenocarcinoma	Cancer derived line	■	■
**CMK**	Acute megakaryocytic leukemia cells	Cancer derived line	■	■
**Fibrobl**	Normal child fibroblast, donor sex: female	Untransformed line	■	
**GM12878**	Lymphoblastoid, CEPH/utah, epstein-barr virus transformed	EBV–transformed Line	■	■
**GM12891**	B-lymphocyte, lymphoblastoid, CEPH/utah, epstein-barr virus transformed	EBV–transformed Line	■	
**GM12892**	B-lymphocyte, lymphoblastoid, CEPH/utah, epstein-barr virus transformed	EBV–transformed Line	■	
**GM19239**	B-lymphocyte, lymphoblastoid, yoruba, nigera, epstein-barr virus transformed	EBV–transformed Line	■	
**GM19240**	B-lymphocyte, lymphoblastoid, yoruba, nigera, epstein-barr virus transformed	EBV–transformed Line	■	
**Haepic**	Human amniotic epithelial cells	Cell line	■	■
**HCF**	Human cardiac fibroblasts	Cell line	■	■
**HCM**	Human cardiac myocytes	Cell line	■	■
**HCPE**	Choroid plexus epithelial cells	Cell line		■
**Hct116**	Colorectal carcinoma	Cancer derived line	■	■
**Heepic**	Human esophageal epithelial cells	Cancer derived line	■	■
**Hek293**	Embryonic kidney	Transformed line	■	■
**HelaS3**	Cervical carcinoma	Cancer derived line	■	■
**Hepatocyte**	Primary human hepatocytes, liver perfused by enzymes to generate single cell suspension	Primary cells	■	
**HepG2**	Liver carcinoma	Cancer derived line	■	■
**Hipepic**	Human iris pigment epithelial cells	Cell line	■	■
**Hl60**	Human promyelocytic leukemia cells	Cancer derived line	■	■
**HMEC**	Human mammary epithelial cells	Cell line	■	■
**Hnpcepic**	Human non-pigment ciliary epithelial cells	Cell line	■	■
**HPAE**	Pulmonary alevolar epithelial cells	Cell line		■
**HRCE**	Human renal cortical epithelial cells (normal)	Cell line	■	■
**HRE**	Human renal epithelial cells (normal)	Cell line	■	■
**Hrpepic**	Human retinal pigment epithelial cells	Cell line	■	■
**HSMM**	Normal human skeletal muscle myoblasts	Cell line	■	
**Hsmmtube**	Normal human skeletal muscle myotubes	Cell line	■	
**Imr90**	Normal human lung fibroblasts	Cell line	■	
**Jurkat**	T lymphoblastoid derived from an acute T cell leukemia	Cancer derived line	■	■
**K562**	Leukemia, 53-year-old female with chronic myelogenous leukemia in terminal blast crises	Cancer derived line	■	■
**Lncap**	Prostate adenocarcinoma	Cancer derived line	■	■
**LncapAndro**	Prostate adenocarcinoma	Cell line	■	
**MCF7**	Mammary gland, adenocarcinoma	Cancer derived line	■	■
**Melano**	Epidermal melanocytes	Cell line	■	
**Nb4**	Acute promyelocytic leukemia cell line	Cancer derived line	■	■
**NHA**	Normal human astrocytes	Cancer derived line	■	■
**NHBE**	Bronchial epithelial cells	Cell line		■
**NHDFneo**	Neonatal human dermal fibroblasts	Cell line	■	■
**Osteobl**	Normal human osteoblasts (nhost)	Cell line	■	
**Panc1**	Pancreatic carcinoma	Cancer derived line	■	■
**PrEC**	Prostate epithelial cell line	Cell line		■
**Progfib**	Fibroblasts, hutchinson-gilford progeria syndrome	Cell line	■	
**Rptec**	Renal proximal tubule epithelial cells	Cell line	■	■
**SAEC**	Small airway epithelial cells	Cell line	■	■
**SKMC**	Human skeletal muscle cells	Cell line	■	■
**Sknsh**	Neuroblastoma	Cancer derived line		■
**Sknshra**	Neuroblastoma cell line, treatment: differentiated with retinoic acid.	Cancer derived line	■	

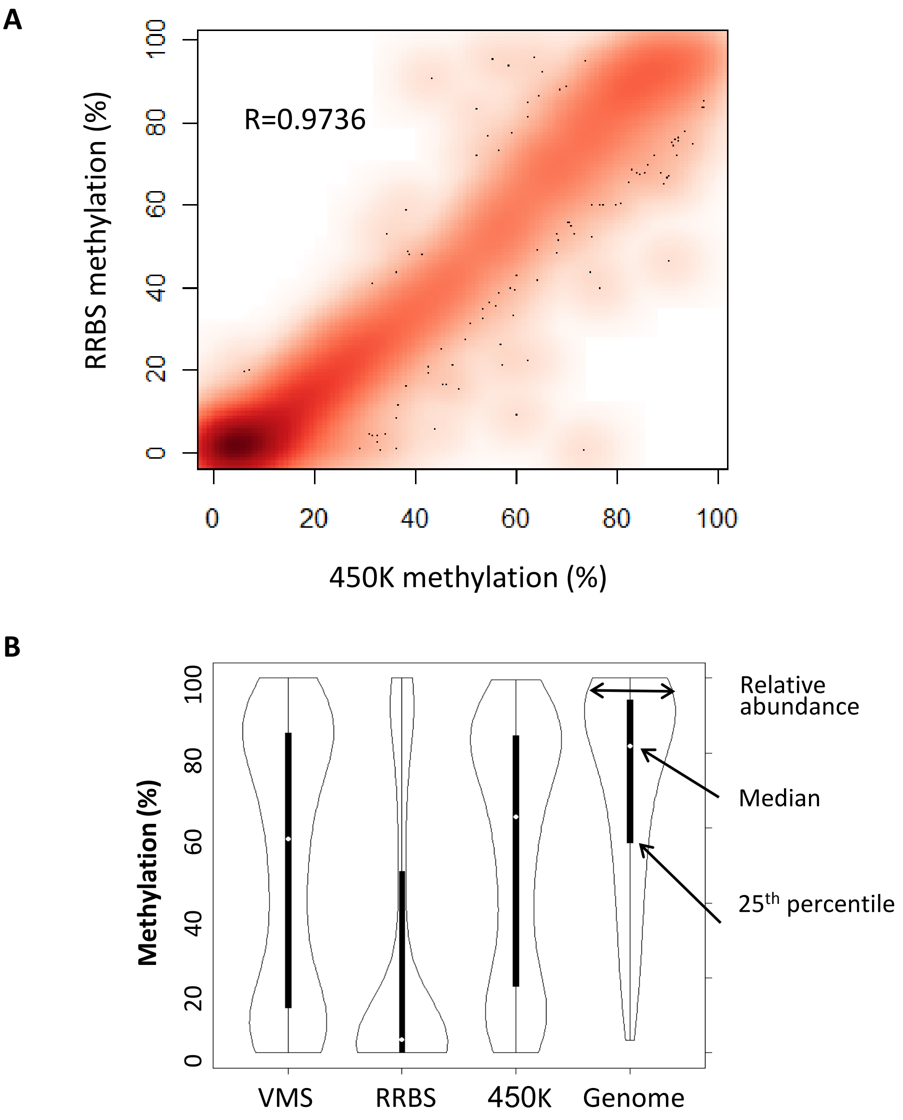

<u>Figure S1</u>. The overall structure of the DNA methylation data. A. Methylation levels of 11,932 CpG sites assessed by reduced representation bisulfite sequencing (RRBS), as function of their methylation levels as assessed by the Inifnium HumanMethylation450 BeadChip assay (450K). The average methylation levels of each site in the 36 cell types that were assessed by the both methylation assays (Table S1) is shown. Linear regression (R) value is indicated. **B.** Distributions of methylation levels in the variable methylation sites (VMS) selected for the study, compared with sites targeted by RRBS, by 450K BeadArray, or sites across the genome (Lister R *et al* Nature 2009 462(7271):315-322). Average methylation of each methylation site across the cell types is shown.

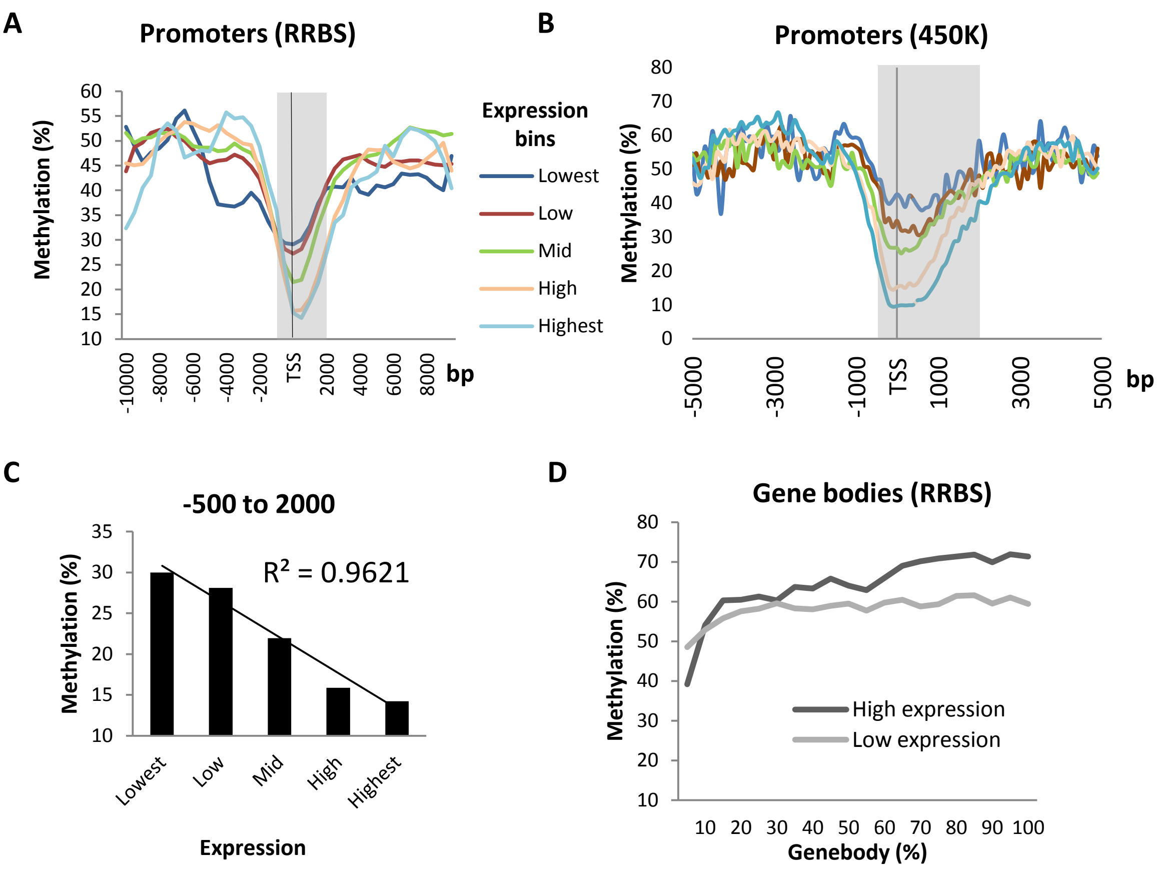

<u>Figure S2</u>. Methylation levels around transcription start sites (TSS) as function of gene expression levels. Human genes were categorized into five expression bins (color code). **A.** Average methylation levels of variable methylation sites (VMSs) across cell types, measured by reduced representation bisulfite sequencing (RRBS). **B.** Average methylation levels of VMSs across cell types, measured by the Inifnium HumanMethylation450 BeadChip (450K). The gray zones in (A) and (B) indicate the -500 to +2000 bp range demonstrating the highest methylation differences between expression bins. **C.** Linear correlation between average methylation levels and expression bins in the -500+2000 range. **D.** Methylation levels in the transcribed portion of the genes (gene bodies) as function of expression levels (highest and lowest gene expression quartiles).

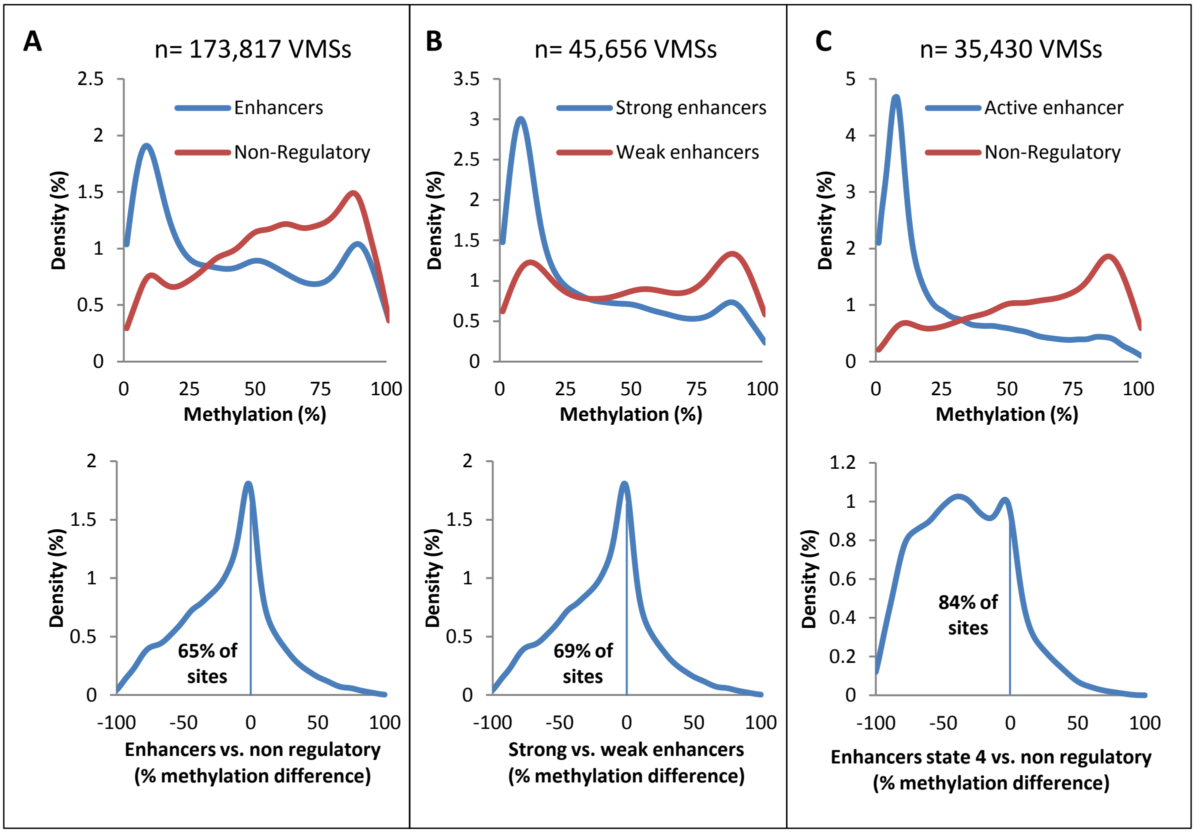

Figure S3. Genomic sites are hypomethylated when marked as enhancer chromatin, compared with the methylation of the same sites in non-regulatory chromatin. Upper panels: Methylation levels of variable methylation sites (VMSs) in cell types where they reside within enhancer chromatin (ChromHMM states 4-7, Ernst *et al* Nature 2011, 473(7345):43-49), versus the methylation of the same sites in cells where they reside in non-regulatory chromatin (ChromHMM states 9-15). Average methylation levels of each site across the cell types carrying the given chromatin blueprints (out of the six cell types for which ChromHMM data were available) are shown. **Lower panels:** The fraction of sites that were hypomethylated when reside in enhancers, compared to their methylation levels in non-regulatory chromatin. **A.** Methylation levels of VMSs residing in enhancers (states 4-7) , versus their methylation in non-regulatory chromatin (states 9-15). **B.** Methylation levels of VMSs residing in strong enhancers (states 4-5), versus their methylation in weak/poised enhancers (states 6-7). **C.** Methylation levels of VMSs residing in enhancer state 4, versus their methylation in non-regulatory chromatin (states 9-15).

A

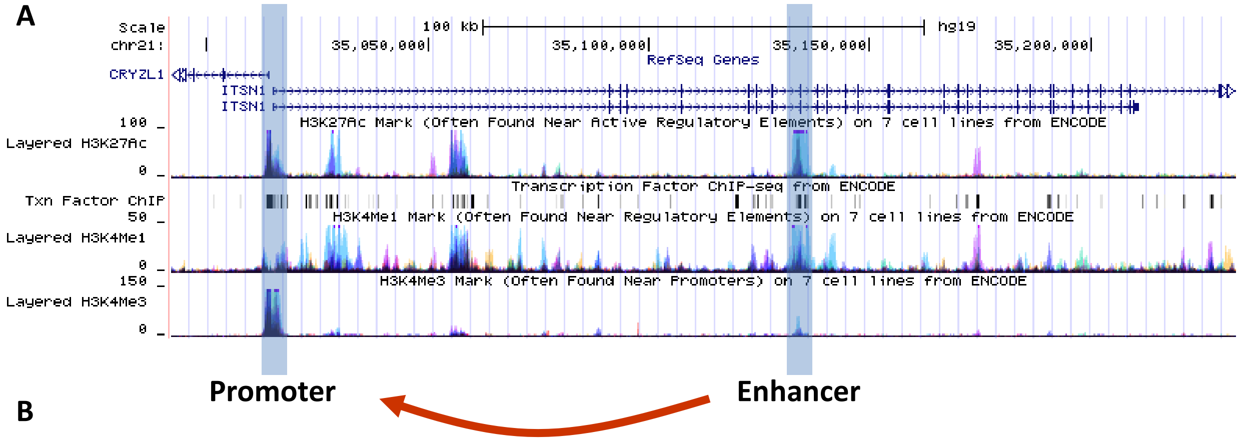

B

Cell type	Interaction (# of sequence reads)
Gm12878	26.5
**HeLa-S3**	**436**
K562	42.5

C

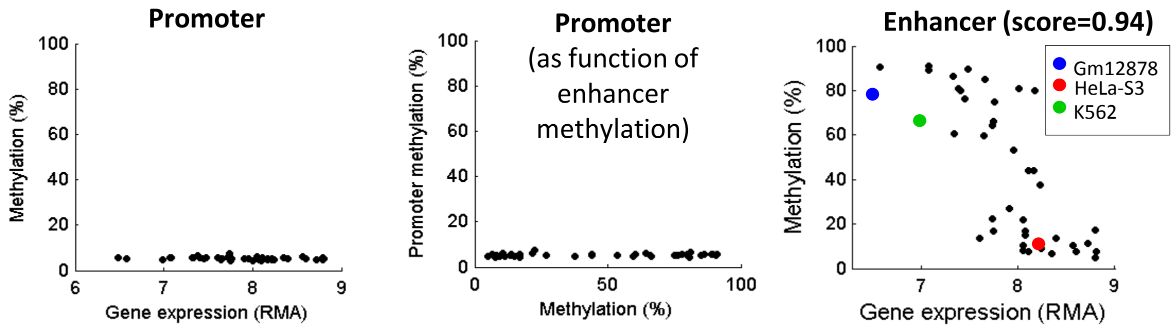

Figure S4. Agreement between methylation-based gene-enhancer pairing and long-range chromatin interactions assessed by the chromosome conformation capture carbon copy (*5C*) technique . A. Genomic map of the *ITSN1* gene region. Relative abundance of regulatory histone marks are shown below the map. Gray boxes mark the regions probed by the 5C assay. **B.** Results of the *5C* assay in 3 cell types, suggesting physical interaction between an enhancer site (marked by the enhancer histone marks H3K27Ac and H3K4Me1) and the *ITSN1* promoter. **C.** Results of the methylation-based assay for the promoter and enhancer sites shown in B-C. **Left:** x-y scatter showing the methylation versus expression levels of the promoter site across the cell types (black dots). **Middle:** methylation of the promoter site versus the methylation of the enhancer across the cell types. **Right:** Methylation versus expression levels of the enhancer site over the cell types (the 3 cell types which also analyzed by the *5C* assay are marked). This site obtained a very high score (0.94) by our assay. Note that in accordance with the methylation-related model of enhancer activity (Figure 3C), physical interactions corresponded with low methylation and high expression (Hela-S3 cells), while methylated cells (Gm12878 and K562) showed low expression and no physical interactions.

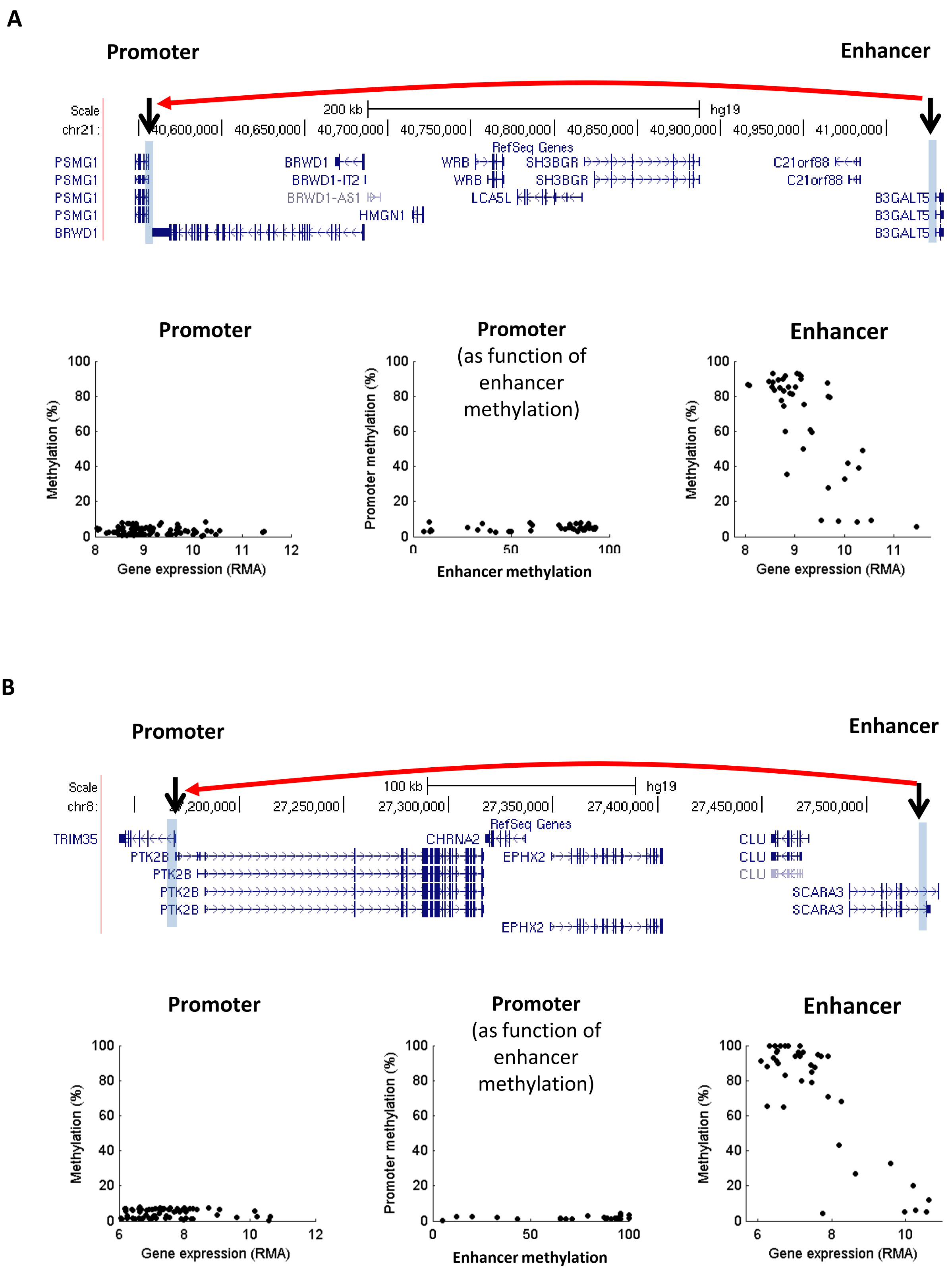

A
Promoter
Enhancer
Scale
chr21:
200 kb
hg19
40,600,000
40,650,000
40,700,000
40,750,000
40,800,000
40,850,000
40,900,000
40,950,000
41,000,000
RefSeq Genes
PSMG1
BRWD1
BRWD1-IT2
BRWD1-AS1
HMGN1
WRB
SH3BGR
LCA5L
C21orf88
B3GALT5
Promoter
Methylation (%)
Gene expression (RMA)
Promoter
(as function of
enhancer
methylation)
Promoter methylation (%)
Enhancer methylation
Enhancer
Methylation (%)
Gene expression (RMA)
B
Promoter
Enhancer
Scale
chr8:
100 kb
hg19
27,250,000
27,300,000
27,350,000
27,400,000
27,450,000
27,500,000
RefSeq Genes
TRIM35
PTK2B
CHRNA2
EPHX2
CLU
SCARA3
Promoter
Methylation (%)
Gene expression (RMA)
Promoter
(as function of
enhancer
methylation)
Promoter methylation (%)
Enhancer methylation
Enhancer
Methylation (%)
Gene expression (RMA)

C

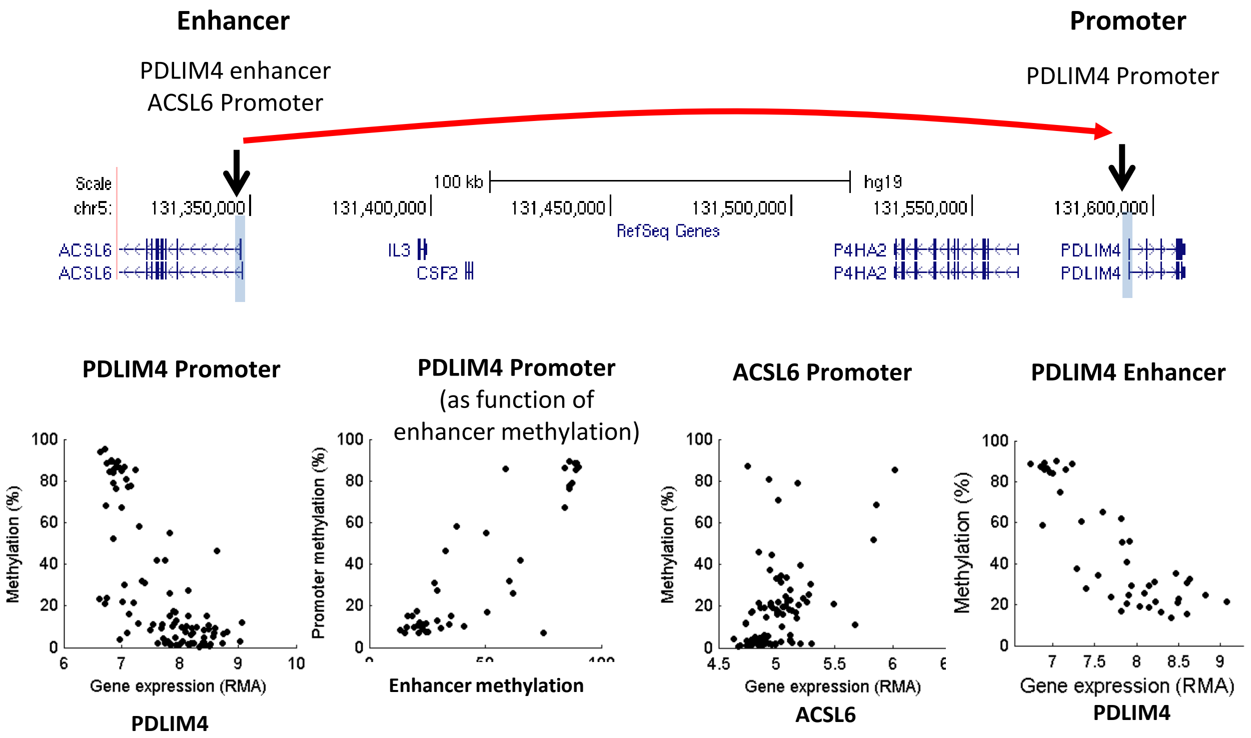

<u>Figure S5</u>. Examples of enhancers located between distal genes (A), within a distal gene (B), or in the promoter of a distal gene (C). The colored areas below the map denote chromatin states in nine cell types. The x-y scatters above the map show methylation of the predicted enhancer site as function of expression levels across the cell types, average methylation of the promoter methylation sites as function of expression levels, or average methylation of the promoter methylation sites as function of methylation of the enhancer site). For the example of an enhancer within a promoter of another gene (C), the methylation of both sites (prompter and enhancer) is compared with the expression of both genes.

Table S2. Normal and cancer cell types.

Normal cell types

Cell	Description	Tissue
**Ag04449**	Fetal buttock/thigh fibroblast	Skin
**Ag04450**	Fetal lung fibroblast	Lung
**Ag09319**	Gum tissue fibroblasts from apparently heathly 24 year old	Gingiva
**AoSMC**	Aortic smooth muscle cells	Heart
**Astrocy**	Astrocytes	Neuroglia
**Bj**	Skin fibroblast	Skin
**Fibrobl**	Normal child fibroblast, donor sex: female	Skin
**GM12878**	Lymphoblastoid, CEPH/utah, epstein-barr virus transformed	Blood
**Hepatocyte**	Primary human hepatocytes	Liver
**HRCE**	Human renal cortical epithelial cells (normal)	Kidney
**HRE**	Human renal epithelial cells (normal)	Kidney
**HSMM**	Normal human skeletal muscle myoblasts	Muscle
**Hsmmtube**	Normal human skeletal muscle myotubes	Muscle
**NHA**	Normal human astrocytes	Neuroglia
**NHBE**	Bronchial epithelial cells	Bronchiole
**Osteobl**	Normal human osteoblasts (nhost)	Bone
**PrEC**	Prostate epithelial cell line	Prostate
**SAEC**	Small airway epithelial cells	Bronchiole

Cancer cell types

Cell	Description	Tissue
**A549**	Epithelial cell line derived from a lung carcinoma tissue	Lung
**Be2_c**	Neuroblastoma	Brain
**Caco2**	Colorectal adenocarcinoma	Colon
**Cmk**	Acute megakaryocytic leukemia cells	Blood
**Hct116**	Colorectal carcinoma	Colon
**Hek293**	Embryonic kidney	Kidney
**Helas3**	Cervical carcinoma	Cervix
**Hepg2**	Liver carcinoma	Liver
**Hl60**	Human promyelocytic leukemia cells	Blood
**Jurkat**	T lymphoblastoid derived from an acute T cell leukemia	Blood
**K562**	Leukemia, 53-year-old female with chronic myelogenous leukemia in terminal blast crises	Blood
**Lncap**	Prostate adenocarcinoma	Prostate
**LncapAndro**	Prostate adenocarcinoma	Prostate
**MCF7**	Mammary gland, adenocarcinoma	Breast
**Nb4**	Acute promyelocytic leukemia cell line	Blood
**Panc1**	Pancreatic carcinoma	Pancreas
**Sknsh**	Neuroblastoma	Brain
**Sknshra**	Neuroblastoma cell line, treatment: differentiated with retinoic acid.	Brain

Table S3. Genes that are hypomethylated and upregulated in lung, breast, and various cancer types.

Gene Symbol	Gene description
**ACP1**	Acid phosphatase 1, soluble
**BCCIP**	BRCA2 and CDKN1A interacting protein
**BRPF1**	Bromodomain and PHD finger containing, 1
**C1orf156**	Chromosome 1 open reading frame 156
**CBX8**	Chromobox homolog 8
**CCT5**	Chaperonin containing TCP1, subunit 5 (epsilon)
**CENPF**	Centromere protein F, 350/400kda (mitosin)
**CENPH**	Centromere protein H
**CENPK**	Centromere protein K
**CHAF1A**	Chromatin assembly factor 1, subunit A (p150)
**CHD1**	Chromodomain helicase DNA binding protein 1
**COCH**	Coagulation factor C homolog, cochlin (limulus polyphemus)
**DCTPP1**	Dctp pyrophosphatase 1
**DNAJB7**	Dnaj (hsp40) homolog, subfamily B, member 7
**DTL**	Denticleless homolog (drosophila)
**EEF1A2**	Eukaryotic translation elongation factor 1 alpha 2
**EME1**	Essential meiotic endonuclease 1 homolog 1 (S. Pombe)
**EXOSC8**	Exosome component 8
**FASTKD3**	FAST kinase domains 3
**FBXO5**	F-box protein 5
**GET4**	Golgi to ER traffic protein 4 homolog (S. Cerevisiae)
**GINS2**	GINS complex subunit 2 (psf2 homolog)
**GTPBP5**	GTP binding protein 5 (putative)
**HDAC1**	Histone deacetylase 1
**HIST1H2AM**	Histone cluster 1, h2am
**HIST1H2BN**	Histone cluster 1, h2bn
**HSPA14**	Heat shock 70kda protein 14
**KIF15**	Kinesin family member 15
**KLHDC4**	Kelch domain containing 4
**LEO1**	Leo1, paf1/RNA polymerase II complex component, homolog (S. Cerevisiae)
**LMNB1**	Lamin B1
**MBTD1**	Mbt domain containing 1
**MCM3AP-AS**	MCM3AP antisense RNA (non-protein coding)
**MCM4**	Minichromosome maintenance complex component 4
**NCAPG**	Non-smc condensin I complex, subunit G
**NVL**	Nuclear vcp-like
**PAXIP1**	PAX interacting (with transcription-activation domain) protein 1
**PCK2**	Phosphoenolpyruvate carboxykinase 2 (mitochondrial)
**PCNT**	Pericentrin
**PNO1**	Partner of NOB1 homolog (S. Cerevisiae)
**PRIM1**	Primase, DNA, polypeptide 1 (49kda)
**PRKDC**	Protein kinase, dna-activated, catalytic polypeptide
**PSMA2**	Proteasome (prosome, macropain) subunit, alpha type, 2
**PUM2**	Pumilio homolog 2 (drosophila)
**RACGAP1**	Rac gtpase activating protein 1
**RRS1**	RRS1 ribosome biogenesis regulator homolog (S. Cerevisiae)
**SCYL3**	Scy1-like 3 (S. Cerevisiae)
**STAT5B**	Signal transducer and activator of transcription 5B
**SUV39H2**	Suppressor of variegation 3-9 homolog 2 (drosophila)
**TAF4**	TAF4 RNA polymerase II, TATA box binding protein (tbp)-associated factor, 135kda
**TFAM**	Transcription factor A, mitochondrial
**TH1L**	Th1-like (drosophila)
**TRIP13**	Thyroid hormone receptor interactor 13
**UHRF1**	Ubiquitin-like with PHD and ring finger domains 1
**UMPS**	Uridine monophosphate synthetase
**USP10**	Ubiquitin specific peptidase 10
**WDHD1**	WD repeat and hmg-box DNA binding protein 1
**ZNF511**	Zinc finger protein 511

Table S4. Enriched gene-ontology (GO) groups among the 207 genes that hypomethylated and upregulated in various cancers compared with various normal cell types.

GO term	Description	# of genes	FDR q-value
GO:0006259	DNA metabolic process	42	3.89E-17
GO:0046483	Heterocycle metabolic process	99	5.35E-17
GO:0006139	Nucleobase-containing compound metabolic process	97	5.17E-17
GO:0006725	Cellular aromatic compound metabolic process	99	4.3E-17
GO:0090304	Nnucleic acid metabolic process	90	3.68E-17
GO:0034641	Cellular nitrogen compound metabolic process	101	4.82E-17
GO:1901360	Organic cyclic compound metabolic process	99	9.33E-16
GO:0006807	Nitrogen compound metabolic process	103	7.59E-15
GO:0044260	Cellular macromolecule metabolic process	116	1.43E-14
GO:0044237	Cellular metabolic process	133	2.26E-13
GO:0006281	DNA repair	23	1.72E-10
GO:0044238	Primary metabolic process	131	4.03E-10
GO:0007049	Cell cycle	30	4.35E-10
GO:0071704	Organic substance metabolic process	132	7.47E-10
GO:0043170	Macromolecule metabolic process	116	1.04E-09
GO:0008152	Metabolic process	135	2.36E-09
GO:0022402	Cell cycle process	38	2.98E-09
GO:0006396	RNA processing	24	3.28E-09
GO:0051276	Chromosome organization	28	9.21E-08
GO:0000278	Mitotic cell cycle	21	1.78E-07
GO:0006974	Response to DNA damage stimulus	26	3.54E-07
GO:0034622	Cellular macromolecular complex assembly	23	3.62E-07
GO:0016070	RNA metabolic process	63	4.21E-07
GO:0034645	Cellular macromolecule biosynthetic process	59	4.37E-06
GO:0006260	DNA replication	17	3.33E-05
GO:0006397	Mrna processing	13	3.43E-05
GO:0022403	Cell cycle phase	22	3.56E-05
GO:0009059	Macromolecule biosynthetic process	61	3.44E-05
GO:0016071	Mrna metabolic process	18	4.79E-05
GO:0006333	Chromatin assembly or disassembly	7	6.75E-05
GO:0006325	Chromatin organization	19	6.59E-05
GO:0034660	Ncrna metabolic process	12	6.89E-05
GO:0044249	Cellular biosynthetic process	69	9.31E-05

<u>Table S5</u>. Genes that are hypomethylated and upregulated in lymphoblastoids derived from acute leukemia versus normal dividing lymphoblastoids, and were also hypomethylated and upregulated in the various cancer versus various normal cell types analysis.

Gene symbol	Gene description
**BAT4**	HLA-B associated transcript 4
**BCCIP**	BRCA2 and CDKN1A interacting protein
**C1orf156**	Chromosome 1 open reading frame 156
**C21orf58**	Chromosome 21 open reading frame 58
**CCT3**	Chaperonin containing TCP1, subunit 3 (gamma)
**CSTF2**	Cleavage stimulation factor, 3' pre-rna, subunit 2, 64kda
**DHPS**	Deoxyhypusine synthase
**DSCC1**	Defective in sister chromatid cohesion 1 homolog (S. Cerevisiae)
**FARSA**	Phenylalanyl-trna synthetase, alpha subunit
**FBXO5**	F-box protein 5
**FKBP4**	FK506 binding protein 4, 59kda
**GPATCH4**	G patch domain containing 4
**HSPA14**	Heat shock 70kda protein 14
**ING5**	Inhibitor of growth family, member 5
**MCM10**	Minichromosome maintenance complex component 10
**MCM3AP-AS**	MCM3AP antisense RNA (non-protein coding)
**MTBP**	Mdm2, transformed 3T3 cell double minute 2, p53 binding protein (mouse) binding protein, 104kda
**NAT10**	N-acetyltransferase 10 (gcn5-related)
**NR2C2AP**	Nuclear receptor 2c2-associated protein
**PCNT**	Pericentrin
**PRAME**	Preferentially expressed antigen in melanoma
**PYGO2**	Pygopus homolog 2 (drosophila)
**RNASEH2A**	Ribonuclease H2, subunit A
**SCYL3**	Scy1-like 3 (S. Cerevisiae)
**SUV39H2**	Suppressor of variegation 3-9 homolog 2 (drosophila)
**TH1L**	Th1-like (drosophila)
**TIMM8A**	Translocase of inner mitochondrial membrane 8 homolog A (yeast)
**UMPS**	Uridine monophosphate synthetase
**WDHD1**	WD repeat and hmg-box DNA binding protein 1
**WDR62**	WD repeat domain 62
**ZNF473**	Zinc finger protein 473
**ZNF552**	Zinc finger protein 552
